# Special Issue “HPV in the Head and Neck Region 2.0”

**DOI:** 10.3390/v15051119

**Published:** 2023-05-06

**Authors:** Tina Dalianis, Christian von Buchwald, Anders Näsman, Stina Syrjanen

**Affiliations:** 1 Department of Oncology-Pathology, Karolinska Institutet, Bioclinicum J6:20, Karolinska University Hospital, 171 64 Stockholm, Sweden; anders.nasman@ki.se; 2 Department of Otorhinolaryngology—Head and Neck Surgery and Audiology, Copenhagen University Hospital, Rigshospitalet, 2100 Copenhagen, Denmark; christian.von.buchvald@regionh.dk; 3 Department of Clinical Pathology, CCK R8:02, Karolinska University Hospital, 171 64 Stockholm, Sweden; 4 Department of Oral Pathology and Radiology, Faculty of Medicine, University of Turku, FI-20014 Turku, Finland; stisyr@utu.fi; 5 Department of Pathology, University of Turku, Turku University Hospital, FI-20014 Turku, Finland

Members of the human papillomavirus (HPV) family have been known for causing cancers and condylomas in the anogenital tract for some time, as reflected by the Nobel Prize in Medicine given to Professor Harald zur Hausen 2008. In the past decades, however, much attention has also been directed to the importance of HPV and diseases in the head and neck region. This is because the cases of HPV-associated head and neck carcinomas are increasing globally. Recently, we presented a Special Issue on “HPV in the head and neck region” which included several contributions to the overall knowledge of HPV in the head and neck region. In this second “Special Issue on HPV in the head and neck region 2.0”, eight original articles, one review, and one systematic review are presented [1,2,3,4,5,6,7,8,9,10].

One of the original articles investigated whether HPV or human polyomaviruses (HPyV) had an aetiological role in the development of adenoid cystic carcinoma (AdCC) in a cohort of 68 diagnosed with AdCC between 2000–2014; no cases with HPyV were detected, while three cases were HPV positive [1]. The authors conclude that neither HPV nor HPyV play a major role in the development of AdCC. However, when scrutinizing the tumour’s disclosing presence of HPV, the diagnosis was more likely to be an HPV-related multiphenotypic carcinoma than AdCC, although a strict subsite location to the sinonasal area was not found in these three tumours [1].

Another of the original papers examined the presence and role of HPV in 48 mucoepidermoid carcinomas (MEC) [2]. HPV was only found in 1/48 cancers, and thus it does not play a major role in this cancer either. This HPV-positive tumour presented high levels of HPV16 E6 and E7 RNA expression and HPV16 was integrated and affected more than 13 genes [2]. The authors concluded that HPV detection in MEC is rare. However, when present, HPV is transcriptionally active, it has a substantial role in altering the host genome.

Three of the original articles describe the prevalence of HPV in locations and various consequences [3,4,5]. One article described the presence of HPV16L1 and HPV16E7 antibodies in the saliva and serum of 39 pregnant women and disclosed that 13 had persistent oral infection, but there was no correlation between a persistent infection and viral antibodies. A significant correlation was found between HPV16L1-specific IgG antibodies in saliva and serum, but not for HPV16L1-specific IgA antibodies [3]. This result was expected as salivary IgGs are mostly serum-derived via mucosal surfaces and gingival fluid, while sIgA, the main immunoglobulin type in saliva, is locally produced. Another article focused on the HPV detection rate in oropharyngeal carcinomas as no previous studies exist on this topic in Chile. HPV was detected in 61.2% of the 49 oropharyngeal carcinomas, the most prevalent genotype being HPV16 (80%). HPV16E6 and E7 transcripts were detectable in 92% and 79% of the HPV16-positive specimens, respectively, [4]. The third article focused on the risk of encountering a second primary cancer (SPC) after presenting an HPV-positive or HPV-negative oropharyngeal cancer (OPSCC) [5]. The authors followed up with 2584 patients diagnosed with OPSCC between 2000–2020, and during the follow-up period, 317 patients were diagnosed with an SPC with a median time interval of 2 years. Patients with HPV+ OPSCC had a significantly longer time to SPC when compared to those with HPV− OPSCC and the former had a significantly better survival as compared to the latter group [5].

A sixth original article investigated the presence of the neutrophile-to-lymphocyte ratio (NLR) as a prognostic marker in HPV+ and HPV− OPSCC [6]. The authors found that an increasing NLR ratio was associated with a worse recurrence-free survival as well as a worse overall survival independent of HPV status [6].

A seventh original article investigated the value of post-treatment neck dissection (ND) after tonsillar and base tongue cancer in the Era of PET-CT, HPV, and p16 [7]. The prevalence and localisations of viable tumour cells in neck lymph nodes were identified by fluorodeoxyglucose positron-emission tomography with computer tomography (FDG PET-CT). FDG PET-CT data were compared with the findings in the pathology report after the ND. The authors concluded that FDG PET-CT, 12 weeks after initial treatment, was useful but not entirely reliable for locating all metastases of HPV+ TSCC/BOTSCC; nevertheless, the data indicate that an ND could be more selectively guided by FDG PET-CT [7].

An eighth original article described attempts to experimentally use various targeted therapies for HPV+ and HPV− tonsillar and base tongue cancer [8]. The authors used 4 cell lines of which three were HPV+ and one HPV-. The study using various targeted therapies showed that especially combining the CDK4/6 inhibitor PD-0332991 with the PI3K inhibitor BYL719 presented synergy and enhanced the decrease in viability considerably, while although dose-dependent responses were obtained with PARP and WEE1 inhibitors (BMN-673 and MK-1775, respectively), synergy was rarely disclosed [8].

The review included in this Special Issue focuses on high-risk HPV-E6 or -E7-based vaccines, based on plasmid, messenger RNA, or peptide, and their present stage of development and testing [9]. In addition, it describes how nanoparticles can be made to target and access cancer cells as well as activate specific immunology pathways besides serving as a delivery vehicle [9].

Finally, the systematic review investigated the presence of HPV in oral cavity squamous cell carcinoma (OCSCC) and included 31 studies comprising 5007 from 24 countries [10]. The overall HPV+ OCSCC prevalence was 6% (95% CI; 3–10%). The authors concluded that HPV in OCSCC is likely not a strong risk factor for OCSCC. Furthermore, upon the detection of HPV, a site misclassification of the mobile tongue with the root of the tongue cannot be excluded [10].

With this summary, we would like to thank all authors for their very informative contributions to this Special Issue. It has been of great interest to follow the most recent events in this relatively new cancer field and hopefully, many of these contributions will also be helpful to both better prevent disease as well as improve tailored treatment.
